# Supplementary material for: Long-Term Survival of Patients with Unresectable Hepatocellular Carcinoma Treated with Lenvatinib in Real-World Clinical Practice
Source: Cancers (Basel). 2025 Feb 1;17(3):479. doi: 10.3390/cancers17030479 (PMC11816230; doi:10.3390/cancers17030479)
Supplement: Supplementary file 1 [file cancers-17-00479-s001.zip › cancers-3362211-supplementary.pdf]

Supplementary Materials

Table S1: OS by post-lenvatinib treatment for HCC

	n	Event	Censored	Median OS (months)[a] (95% CI)
No subsequent treatment	202	126	76	20.3 (17.0, 23.5)
Any subsequent treatments	208	129	79	26.1 (23.7, 27.4)
Chemotherapy	150	99	51	24.7 (23.0, 26.9)
TACE	71	44	27	24.3 (20.0, 30.3)
HAIC	33	24	9	19.2 (14.1, 27.1)
Percutaneous radiofrequency ablation	18	5	13	NR (26.1, -)
Radiotherapy	17	14	3	25.0 (13.9, 35.1)
Surgery	7	3	4	NR (26.2, -)

[a] Estimated by the Kaplan–Meier method

CI, confidence interval; HAIC, hepatic arterial infusion chemotherapy; HCC, hepatocellular carcinoma; NR, not reached; OS, overall survival; TACE, transcatheter arterial chemoembolization
